# Integrating Cross-Modal Semantic Learning with Generative Models for Gesture Recognition

**DOI:** 10.3390/s25185783

**Published:** 2025-09-17

**Authors:** Shuangjiao Zhai, Zixin Dai, Zanxia Jin, Pinle Qin, Jianchao Zeng

**Affiliations:** School of Computer Science and Technology, North University of China, Taiyuan 030051, China

**Keywords:** gesture recognition, radio frequency sensing, cross-modal semantic learning, generative models

## Abstract

Radio frequency (RF)-based human activity sensing is an essential component of ubiquitous computing, with WiFi sensing providing a practical and low-cost solution for gesture and activity recognition. However, challenges such as manual data collection, multipath interference, and poor cross-domain generalization hinder real-world deployment. Existing data augmentation approaches often neglect the biomechanical structure underlying RF signals. To address these limitations, we present CM-GR, a cross-modal gesture recognition framework that integrates semantic learning with generative modeling. CM-GR leverages 3D skeletal points extracted from vision data as semantic priors to guide the synthesis of realistic WiFi signals, thereby incorporating biomechanical constraints without requiring extensive manual labeling. In addition, dynamic conditional vectors are constructed from inter-subject skeletal differences, enabling user-specific WiFi data generation without the need for dedicated data collection and annotation for each new user. Extensive experiments on the public MM-Fi dataset and our SelfSet dataset demonstrate that CM-GR substantially improves the cross-subject gesture recognition accuracy, achieving gains of up to 10.26% and 9.5%, respectively. These results confirm the effectiveness of CM-GR in synthesizing personalized WiFi data and highlight its potential for robust and scalable gesture recognition in practical settings.

## 1. Introduction

Radio frequency (RF) signals are increasingly recognized as an important medium for the integration of sensing and communication in ubiquitous computing systems. RF signals such as WiFi and RFID not only carry communication data but also embed environmental information through their propagation behaviors. With the rapid expansion of the Internet of Things (IoT), integrating RF-based sensing into everyday environments has become a key objective for the intelligent and unobtrusive monitoring of human activities. Phenomena such as reflection, refraction, and diffraction, produced by interactions with surrounding objects and the human body, encode contextual cues that can be exploited for ambient sensing. This dual role positions RF signals as a fundamental enabler for systems that integrate communication and sensing, thereby advancing pervasive and intelligent applications.

WiFi is particularly attractive for human sensing because of its wide deployment, cost-effectiveness, and non-intrusive nature. Human movements influence WiFi signals during transmission, and these changes can be captured at the physical layer through measurements such as the received signal strength indicator (RSSI) and channel state information (CSI). By analyzing amplitude and phase variations through either handcrafted features or machine learning models, WiFi sensing has been successfully applied to tasks such as indoor localization, gesture recognition, and vital sign monitoring [1]. These systems can operate on commodity edge devices, enabling real-time, low-cost, and privacy-preserving applications in areas such as smart homes and healthcare. For instance, WiFi sensing has been explored for activity monitoring in daily life, intelligent home automation, and contactless health monitoring, including fall detection for elderly individuals [2]. Among these applications, gesture recognition has drawn particular interest because of its potential to support natural and intuitive human–computer interaction [3].

Despite these promising applications, reliable WiFi-based gesture recognition remains difficult in real-world environments. WiFi signals are highly sensitive to environmental changes, with static obstacles (e.g., walls and furniture) and dynamic factors (e.g., human interference) often distorting propagation paths. These distortions often cause the recognition accuracy to degrade when the system encounters new environments or unseen subjects, a challenge widely known as cross-domain sensing [4,5]. This paper specifically addresses cross-subject sensing, where a model must generalize to new, unseen subjects [6]. Addressing this challenge typically requires repeated data collection and labeling for each new user, which is labor-intensive, time-consuming, and impractical for large-scale deployment. Furthermore, the collected data can be inconsistent due to furniture layouts, device statuses, and other environmental factors, resulting in unstable quality that undermines robustness and scalability [7]. This motivates the development of efficient and targeted strategies for generating user-specific WiFi sensing data without extensive manual effort.

Existing methods for WiFi data generation fall into three categories. The first employs augmentation techniques inspired by computer vision, such as flipping and cropping [8,9]. These methods improve the data diversity but rely entirely on existing samples and cannot capture user-specific variations. The second direction uses neural networks to model RF propagation [10], which provides physical interpretability but struggles in dynamic or cluttered environments. The third applies generative models such as generative adversarial networks (GANs) and diffusion approaches [11,12,13] to synthesize WiFi data. While these models enrich training samples, they are limited by the inherently low spatial resolution of WiFi signals. As a result, they fail to encode biomechanical traits and typically produce data with low subject specificity and weak generalization. These shortcomings become particularly problematic when little or no target user data are available.

In this work, we introduce CM-GR, a cross-modal generative framework that addresses these limitations by leveraging semantic priors from vision to guide WiFi data synthesis. Unlike single-modality generative methods, CM-GR exploits 3D skeletal points extracted from video as semantic guidance, embedding biomechanical constraints into the generation process. This design enables the framework to produce user-specific WiFi data without requiring explicit data collection for each target user. To further enhance personalization, CM-GR constructs conditional vectors based on inter-user skeletal differences, which support controllable and user-adaptive synthesis. By combining cross-modal semantic learning with generative modeling, CM-GR ensures both fidelity and personalization, thereby advancing WiFi-based gesture recognition toward scalable and zero-shot deployment.

The main contributions of this paper are summarized as follows:

- We propose CM-GR, a cross-modal gesture recognition framework that synthesizes user-specific WiFi data guided by visual 3D skeletal points.
- We design conditional vectors derived from skeletal semantics to introduce biomechanical constraints into WiFi data generation, enabling personalized synthesis without additional user-specific data collection.
- We develop a dual-stream adversarial architecture with bidirectional domain translation that improves both the fidelity and generalization of generated WiFi data.
- Extensive experiments on the MM-Fi and SelfSet datasets demonstrate that CM-GR significantly outperforms existing methods in cross-subject gesture recognition, improving the accuracy by 10.26% and 9.5%, respectively.

## 2. Background

Generative models aim to learn the underlying data distribution and produce samples that closely resemble real-world observations. Among these, GANs and diffusion models are two leading approaches that are widely used for high-fidelity data synthesis. This section compares the two approaches in terms of their generative processes, controllability (i.e., ability to condition on target-specific semantics), and computational costs, with the goal of evaluating their applicability to RF-based gesture synthesis. The analysis provides insights into which model better supports the personalized, efficient data generation required by the CM-GR framework.

### 2.1. Generative Adversarial Networks (GANs)

GANs are based on adversarial training involving two neural networks. The generator *G* attempts to produce data that mimic the real distribution $P_{data}$, and the discriminator *D* aims to distinguish real samples from those generated. Their interaction is formalized as a minimax optimization problem:

$$\underset{G}{min}\underset{D}{max}E_{x∼P_{data}\left(x\right)}\left[logD(x)\right]+E_{z∼P_{z}\left(z\right)}\left[log(1-D(G(z)))\right]$$

where $P_{z}$ represents a predefined noise distribution. Although GANs are effective in generating realistic samples, they generally lack semantic control over the output, which limits their ability to produce user-specific data.

**Figure 1:**
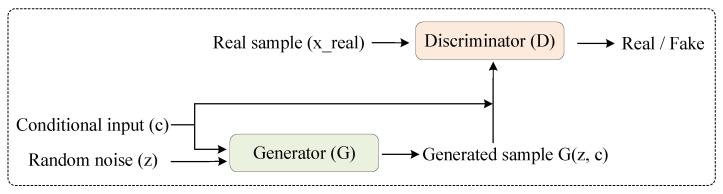
Structure of a conditional GAN (CGAN), where both the generator and discriminator are guided by conditional inputs to enable controlled sample generation.

As shown in Figure 1, CGANs extend GANs by conditioning both the generator and discriminator on conditional inputs during training. In CGANs, the generator receives not only random noise but also an additional conditional input, which jointly guides the generation process to produce attribute-specific samples.

Due to their architectural simplicity and fast inference speeds, GAN-based models are particularly well suited for deployment on resource-constrained platforms. In scenarios that demand low latency and limited computational overhead, GANs offer a favorable trade-off between performance and efficiency. These practical advantages support their integration into the CM-GR framework proposed in this study, where real-time gesture recognition on embedded RF sensing devices is a key objective.

### 2.2. Diffusion Models

**Figure 2:**
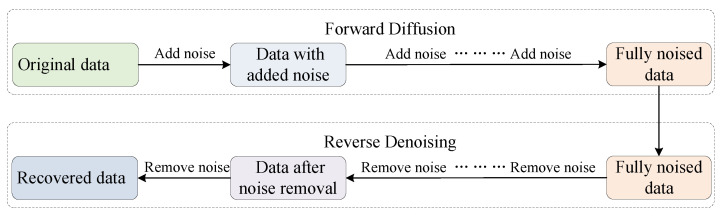
Schematic of the diffusion model workflow, where the forward phase incrementally corrupts the data with noise and the reverse phase restores them through learned denoising steps.

Diffusion models emulate a natural diffusion process by adding noise to the data over several steps and then learning to reverse this process. As shown in Figure 2, the forward diffusion phase introduces noise progressively using techniques such as Markov chains or stochastic differential equations. The reverse denoising phase then reconstructs the original data by learning to remove noise step by step. This bidirectional process facilitates the generation of realistic data and yields more stable training than conventional GANs.

Despite their advantages, diffusion models require a large number of inference steps, which significantly increases the computational costs. This constraint makes them less suitable for applications involving real-time processing or resource-limited devices. In RF-based sensing scenarios, where a rapid response and on-device processing are essential, the high complexity of diffusion models becomes a limiting factor.

These generative methods form the foundation of the CM-GR approach. By combining adversarial generation with cross-modal semantic learning, where visual 3D skeletal points are exploited as semantic priors to guide the synthesis of WiFi signals, the framework aligns heterogeneous modalities and transfers biomechanical information from vision to RF sensing. This cross-modal mechanism ensures that the generated WiFi data not only preserve gesture semantics but also adapt to user-specific motion traits, thereby addressing the challenge of recognizing gestures across different subjects using multimodal data.

## 3. Related Work

This section reviews related work across three areas that underpin the proposed CM-GR framework. First, we survey gesture recognition approaches that utilize vision, inertial sensors, and RF-based systems. Second, we examine multimodal learning techniques that fuse heterogeneous data sources to improve the robustness and generalization. Third, we explore generative modeling frameworks—particularly GANs and diffusion models—for data augmentation and cross-domain synthesis.

### 3.1. Gesture Recognition

Gesture recognition has been studied across multiple sensing modalities, each offering distinct trade-offs in terms of resolution, usability, and robustness. Vision-based methods typically use RGB or depth video to extract rich spatial and temporal features [14,15,16,17]. These methods benefit from a high spatial resolution and contextual awareness, enabling the detailed modeling of skeletal movements and motion trajectories. However, they remain sensitive to lighting variability, background clutter, and privacy constraints [18,19,20,21]. Sensor-based techniques, such as those using inertial measurement units (IMUs) or wearable devices, provide resilience in dynamic or visually obstructed environments [22,23,24]. By capturing motion signals like acceleration and angular velocities directly, these approaches achieve high recognition accuracy under varying conditions. Their main drawback lies in the need for users to wear specific devices, which may reduce their convenience and real-world applicability [25,26]. RF-based gesture recognition, especially using WiFi CSI, offers a promising device-free alternative that preserves user privacy [27,28,29]. By detecting signal reflections caused by human motion, RF sensing supports contactless recognition. Nonetheless, it suffers from a low spatial resolution and is affected by environmental changes such as multipath interference, limiting its generalization across users and locations [30,31].

### 3.2. Multimodal Learning

Multimodal learning integrates complementary information from multiple sensing sources to improve model performance [32]. By fusing vision, auditory, and RF data, multimodal frameworks address the limitations inherent to single modalities and offer a more comprehensive understanding of human activity [33]. In gesture recognition, combining vision and RF modalities is particularly effective. Vision data provide high-resolution spatial details and dynamic motion cues, while RF signals ensure temporal consistency and robustness to occlusion [34]. Recent efforts have focused on feature-level fusion strategies. Chen et al. [35] proposed a method that first extracts features independently from WiFi and video modalities and then uses convolution and cross-attention mechanisms to learn dynamic weights between them; finally, it fuses these features to improve the recognition accuracy in complex environments. In semi-supervised settings, Lu et al. [36] introduced a framework that transfers discriminative knowledge from labeled video segments to unlabeled WiFi signals to enable automatic annotation. Cross-modal knowledge distillation has also been explored. Sheng et al. [37] employed a teacher–student model where the teacher leverages visual information to guide the student in learning from WiFi signals, thereby maximizing the prediction accuracy.

Although these approaches have shown progress in shallow feature alignment and domain transfer, deeper semantic-level knowledge transfer remains underexplored [38]. The need for the improved modeling of high-level behavioral semantics across modalities motivates our proposed CM-GR framework, which integrates semantic priors into the generative process for more effective cross-subject adaptation.

### 3.3. Generative Models

Generative models have gained significant attention for their ability to synthesize realistic data and augment training datasets, especially in domains where data collection is costly or constrained. Among these, GANs and diffusion models represent two prominent paradigms [39]. GANs generate new samples by training a generator to produce outputs that can fool a discriminator trained to distinguish real from synthetic data. In gesture recognition, GANs have been applied to synthesize motion sequences [40], augment sensor signals [41], or transfer gesture styles across users [11]. For example, CsiGAN demonstrates the feasibility of generating WiFi CSI data for improved activity recognition [12]. However, traditional GANs often struggle with semantic control and mode collapse, particularly in cross-subject tasks requiring personalized signal generation. Diffusion models approach the generative task differently. They simulate a physical process of gradually adding noise to the data over multiple steps; they then learn to reverse this process to reconstruct realistic samples. These models offer high sample fidelity and have shown strong performance in vision and audio synthesis. RF-Diffusion applies this principle to RF signal generation, achieving impressive results in signal reconstruction [13]. Despite their effectiveness, diffusion models are computationally intensive due to the iterative denoising process, making them less suitable for deployment in real-time or edge computing environments like WiFi routers.

While GANs and diffusion models offer valuable tools for data generation, few works in the field of RF sensing incorporate cross-modal priors such as visual semantics into the generative process. This gap inspires the CM-GR framework, which combines adversarial training with multimodal supervision to synthesize user-specific RF data for improved cross-subject gesture recognition.

## 4. Our Approach

This section presents an overview and the detailed design of the proposed CM-GR framework. It introduces four core modules, namely the data collection module, the preprocessing module, the data generation module, and the gesture recognition module. These modules will be described in the following subsections.

### 4.1. System Overview

**Figure 3:**
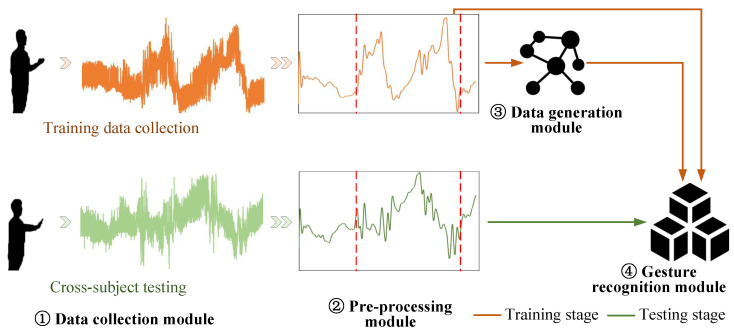
Overview of the proposed CM-GR framework for cross-subject gesture recognition. The system begins by collecting raw WiFi gesture data, followed by denoising and localization to reduce noise and mitigate multipath interference. A gesture recognition model is trained on the cleaned data, and generalization to unseen users is improved by synthesizing user-specific samples through adversarial data generation.

The proposed CM-GR framework is designed to address the challenges of cross-subject gesture recognition using WiFi sensing data. As shown in Figure 3, the framework consists of four core components.

- Data Collection Module: The data collection module plays a key role in capturing raw CSI through a multi-antenna WiFi transceiver. It is responsible for constructing the dataset containing gesture actions, using a miniature computer to gather data across various environments. This foundational step ensures that the system captures a wide variety of gesture patterns, which form the core for further processing.
- Data Preprocessing Module: The data preprocessing module prepares the collected data by applying advanced denoising and localization techniques. These operations aim to remove environmental noise and multipath interference, ensuring that the data are clean and standardized for subsequent analysis. This step is crucial in improving the accuracy and consistency of the gesture recognition model.
- Gesture Recognition Module: Once the data are preprocessed, the gesture recognition module uses them to train a highly accurate gesture recognition model. This module is crucial in interpreting the processed WiFi data and classifying gestures with high precision. However, when deployed to new users, the pretrained model’s representation space struggles to adapt to individual differences in aspects such as height, body shape, and behavior, leading to performance degradation.
- Data Generation Module: To overcome the challenges associated with limited data for new users, the data generation module uses adversarial synthesis to generate high-quality target-specific WiFi sensing data. This process is crucial in enhancing model robustness in cross-subject recognition tasks. The module enables the generation of new data samples without the need for specialized data acquisition from each new user.

### 4.2. Preprocessing Module

Gesture recognition relies on CSI data obtained from WiFi network cards. The physical layer characteristics of CSI are constrained by hardware limitations, leading to non-stationary noise interference. Without addressing this noise, the accuracy and robustness of gesture recognition systems would be significantly compromised. Experimental analysis reveals that environmental disturbances predominantly introduce high-frequency noise, whereas gesture-induced variations occur at lower frequencies. This distinction underscores the necessity of effective signal separation. Therefore, the first step involves filtering out high-frequency noise across all subcarriers. The filtered data are then normalized to maintain consistency across different input samples. Finally, gesture-relevant segments are localized and extracted for subsequent processing.

#### 4.2.1. Signal Denoising

To enhance the quality of CSI data for gesture recognition, it is crucial to filter out high-frequency noise caused by environmental interference. Gesture-induced signal variations typically occur at lower frequencies, while noise tends to dominate higher frequencies. To address this issue, a Butterworth filter is applied across all subcarriers to preserve gesture-relevant low-frequency signals while suppressing irrelevant high-frequency noise. For a carrier frequency of 5.825 GHz, the effective velocity of a gesture signal with an 80 Hz frequency is estimated to be about 2.06 m/s after considering the round-trip propagation path [11]. Therefore, the cut-off frequency of the Butterworth filter is set according to

$$\omega_{s}=\frac{80}{f_{n}/2}$$

where the sampling rate $f_{n}$ is fixed at 1000 in our experiments. This cut-off value is uniformly applied to all subcarriers to ensure consistency across CSI streams. The choice of 80 Hz corresponds to the upper bound of human gesture dynamics, which allows the filtering process to retain gesture-related variations while effectively removing high-frequency interference. Following denoising, the CSI data are standardized to maintain a consistent amplitude across all samples. Each feature is normalized to have zero mean and unit variance, thereby mitigating bias caused by varying feature scales. This standardization step not only improves data comparability but also optimizes the input for downstream machine learning tasks, ultimately enhancing the model performance.

#### 4.2.2. Gesture Localization

Gesture localization is a critical step for accurate gesture recognition, as it allows the system to identify and focus on the relevant segments of the gesture. This is achieved by applying a sliding window approach, where the variance $v_{i}$ of the signal within each window is computed as

$$v_{i}=var\left(x_{i},x_{i+1},...,x_{i+L-1}\right)$$

where $x_{i}$ represents the data points within a window of length *L*. The threshold μ for variance is then calculated as the mean of the variances across all windows:

$$\mu =\frac{1}{N}\sum_{i=1}^{N}v_{i}$$

where *N* is the total number of windows in the dataset. To detect the gesture’s start and end points, the system identifies when the variance exceeds the threshold for a predefined number of consecutive windows. If the variance remains above the threshold for *T* consecutive windows, the first point is marked as the start of the gesture. If the variance falls below the threshold for *T* consecutive windows, the first point is marked as the end of the gesture. This approach effectively isolates the gesture-related data, enabling more precise and reliable gesture recognition by focusing on the signal fluctuations corresponding to the gesture.

### 4.3. Data Generation Module

**Figure 4:**
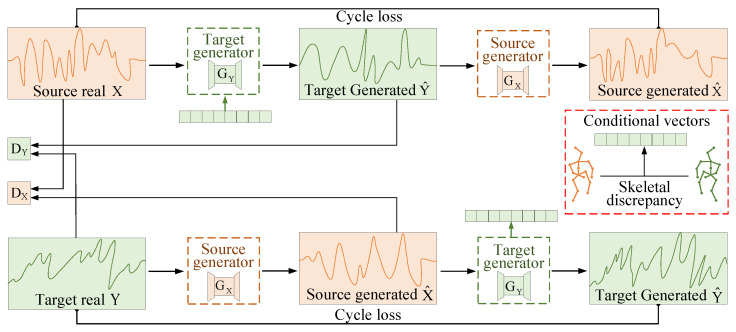
Cross-modal semantic learning-based WiFi data generation. The source-domain data *X* are converted to $\hat{Y}$ by generator $G_{Y}$ with conditional guidance, and discriminator $D_{Y}$ distinguishes *Y* from $\hat{Y}$. Conversely, generator $G_{X}$ maps Y to $\hat{X}$, and discriminator $D_{X}$ distinguishes *X* from $\hat{X}$. Conditional vectors constructed from inter-subject skeletal discrepancies provide semantic guidance for this process.

As shown in Figure 4, the data generation module adopts a dual-generator, dual-discriminator architecture that enables bidirectional feature translation between the source and target domains. This design facilitates the mutual generation of realistic and domain-adaptive WiFi sensing data. Specifically, source-domain WiFi inputs are transformed by the target-domain generator into target-specific representations, while the source-domain generator reconstructs source-like data from the target domain. To guide the generation process toward user-specific motion patterns, the module integrates cross-modal conditional vectors derived from skeletal joint kinematics in vision data. These vectors are constructed from inter-subject skeletal discrepancies and encode biomechanical priors into the signal domain, as described in detail in Section 4.3.3. This design enables the system to synthesize gesture data for unseen users without requiring the dedicated collection of user-specific RF recordings. Instead, the system leverages unlabeled gestures that occur naturally during users’ routine behavior to guide the generation process. This cross-modal semantic learning mechanism is essential in enabling zero-shot generalization. To further improve the fidelity and semantic consistency of the generated data, the module incorporates adversarial loss, cycle consistency loss, and conditional loss. The conditional loss enforces alignment between visual priors and generated RF patterns, ensuring that gesture semantics are preserved during generation. By synthesizing signals that reflect user-specific biomechanics, the module improves the recognition accuracy, reduces the annotation costs, and supports scalable deployment across unseen subjects and environments.

#### 4.3.1. Signal Generator

The generator comprises two components, namely the target-domain generator $G_{Y}$ and the source-domain generator $G_{X}$. As illustrated in Figure 4, $G_{Y}$ maps source-domain WiFi sensing data *X* to synthesized target-domain representations $\hat{Y}$, while $G_{X}$ reconstructs source-like data $\hat{X}$ from $\hat{Y}$. To ensure that the generated target data $\hat{Y}$ preserve gesture-discriminative information from the original input *X*, a cycle consistency loss is applied between X and $\hat{X}$.

This generator design enables personalized gesture synthesis without requiring labeled data from the target user. During training, gesture samples from the source domain are paired with unlabeled, naturally occurring motion patterns from the target domain. The model learns to produce user-specific WiFi data conditioned on domain priors, thus supporting zero-shot generalization in cross-subject recognition scenarios.

As shown in Table 1, the generator architecture adopts an encoder–decoder structure consisting of a downsampling module, a bottleneck composed of residual blocks, and an upsampling module. This design facilitates the extraction of spatial–temporal features while preserving high-level semantic representations, which are essential for generating gesture-aware WiFi signals.

To begin with, the downsampling module consists of convolutional layers, activation functions, and normalization operations. Its purpose is to reduce the dimensionality of the input WiFi signals while capturing key gesture-related features such as the phase, amplitude, and frequency. These condensed features retain the essential dynamics of human motion and provide a discriminative foundation for subsequent processing. Building on these features, the intermediate module leverages a series of residual blocks to model both local and global dependencies in the signal. The residual connections maintain signal continuity, while deeper layers abstract gesture semantics. This design is particularly effective for WiFi sensing, where subtle changes in spatial–temporal patterns encode user intent. In the final stage, the upsampling module uses transposed convolution and normalization to reconstruct the feature maps to their original resolution. This process preserves the spatial integrity of the generated signals and enables high-resolution gesture representations suitable for recognition.

#### 4.3.2. Signal Discriminator

To enforce domain alignment and enhance the generation quality in both directions, the model employs two domain-specific discriminators. One is responsible for the source domain and the other for the target domain. Each discriminator is trained to determine whether a WiFi sample is real or generated within its respective domain. In addition to evaluating authenticity, the discriminators guide the generators to preserve gesture-relevant features during synthesis. The training process follows an adversarial strategy where discriminators distinguish synthetic from real data and generators aim to produce indistinguishable samples. Training proceeds until the discriminators can no longer reliably differentiate between real and generated WiFi signals.

As shown in Table 2, the discriminator consists of a series of convolutional layers (Conv2D), each followed by normalization and activation operations. This layered structure enables the effective extraction of fine-grained gesture patterns from WiFi signals. Through hierarchical convolution, the model transforms spatial features into abstract representations, ultimately encoded as an N×N response map that captures localized authenticity. A patch-based classification strategy is employed to improve the sensitivity of the discriminator to subtle spatial inconsistencies in the generated data. This design is particularly well suited for RF sensing, where gesture-induced signal variations are often sparse and spatially localized. The discriminator thereby learns to assess the fidelity of the generated samples at a fine-grained level, increasing its overall discrimination accuracy. Throughout adversarial training, the generator continuously refines its outputs to resemble real data, while the discriminator sharpens its ability to detect synthetic patterns. This adversarial feedback loop gradually aligns the synthesized WiFi signals with real samples in both structural and distributional properties. As a result, the model produces high-quality, gesture-representative data for cross-subject recognition tasks without requiring additional target user annotations.

#### 4.3.3. Cross-Modal Conditional Vector

Using only WiFi data to generate gesture samples for target users limits data diversity and restricts the modeling of user-specific traits, including the motion amplitude and execution range. This limitation stems from the dependence of WiFi sensing on signal reflections, where subtle motion variations are frequently masked by multipath effects. Furthermore, the inherently low spatial resolution of RF signals restricts the ability to distinguish fine-grained individual differences. To address these challenges, we introduce cross-modal conditional vectors derived from vision data. The visual modality provides a high spatial resolution and rich contextual cues, including skeletal joint positions, motion trajectories, and velocity changes. These cues act as biomechanical priors that guide the generative process, preserving gesture semantics and enabling the synthesis of user-adaptive RF data without additional WiFi recordings from the target subject. At the core of this design are cross-modal conditional vectors, which capture gesture dynamics and user-specific traits to provide essential guidance for generation.

**Figure 5:**
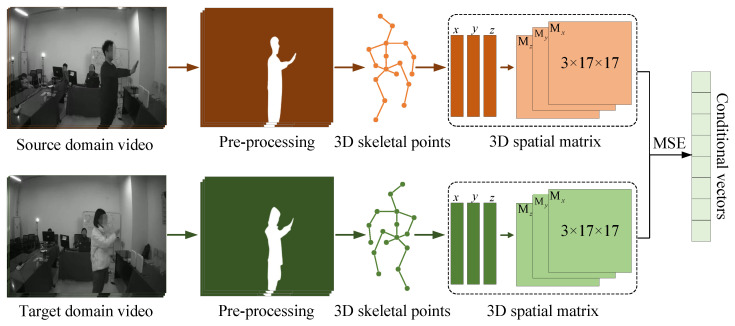
Overview of the pipeline that derives conditional vectors from video data, including preprocessing, skeletal keypoint extraction, and spatial representation.

As illustrated in Figure 5, the construction of effective cross-modal conditional vectors begins with the extraction of meaningful gesture segments from video data. To reduce the computational cost and suppress background noise, RGB frames are first converted to grayscale. Human silhouettes are then segmented using the $U^{2}$-Net model [42], which provides accurate foreground masks for subsequent analysis. Since gestures are primarily defined by temporal motion, we apply an optical flow algorithm to identify the onset and offset of each action, thereby producing temporally precise gesture clips. From these segments, skeletal keypoints are estimated using the VideoPose3D method [43], which employs temporal convolutional networks to capture the dynamics of 2D keypoints over time while remaining computationally efficient. The resulting skeletal points are projected into a 3D spatial matrix that encodes the user’s body configuration during gesture execution, preserving fine-grained skeletal structures and motion patterns. To quantify user-specific differences, we compute the mean squared error (MSE) between spatial matrices across the source and target domains. These matrices are then embedded as conditional vectors, which encapsulate both gesture dynamics and user-specific traits. Serving as cross-modal guidance, these conditional vectors provide a discriminative basis for subsequent generation and alignment within the proposed framework.

#### 4.3.4. Loss Function

A well-designed loss function is essential in guiding effective training and ensuring high-fidelity cross-domain WiFi data generation. In the CM-GR framework, the total loss consists of three components: adversarial loss, cycle consistency loss, and conditional vector loss. Each component targets a specific challenge in cross-subject gesture synthesis, promoting realism, semantic consistency, and user-specific adaptation.

Adversarial Loss. The adversarial loss forms the foundation of the generative process. It encourages the generators to produce WiFi data that cannot be distinguished from real samples, while the discriminators learn to identify synthetic inputs. The adversarial objective consists of two complementary terms, reflecting forward and backward translations across domains. The forward term guides the generation of target-domain data from source inputs, while the backward term ensures that the generated data can be reconstructed back to the source domain. This bidirectional formulation supports consistent and realistic mapping between heterogeneous signal spaces. The formal definition of the adversarial loss is given below:

$$L_{GAN}\left(G_{Y},D_{Y},X,Y\right)=E_{Y∼P(Y)}\left[logD_{Y}\left(Y\right)\right]+E_{X∼P(X)}\left[log(1-D_{Y}\left(G_{Y}\left(X\right)\right))\right]$$

$$L_{GAN}\left(G_{X},D_{X},X,Y\right)=E_{X∼P(X)}\left[logD_{X}\left(X\right)\right]+E_{Y∼P(Y)}\left[log(1-D_{X}\left(G_{X}\left(Y\right)\right))\right]$$

In this formulation, $L_{GAN}\left(G_{Y},D_{Y},X,Y\right)$ and $L_{GAN}\left(G_{X},D_{X},X,Y\right)$ represent the forward and backward adversarial losses, respectively. Here, $G_{X}$ and $G_{Y}$ denote the generators responsible for translating between the source and target WiFi domains, while $D_{X}$ and $D_{Y}$ are the corresponding discriminators. The terms P(X) and P(Y) indicate the data distributions of the source and target domains.

Cycle Consistency Loss. To preserve semantic coherence during bidirectional domain translation, a cycle consistency loss is incorporated. This loss ensures that the original gesture information is retained after data undergo sequential mappings between the source and target domains. Specifically, the source-domain data *X* are first transformed by the target-domain generator $G_{Y}$ into $\hat{Y}=\left(G_{Y}\left(X\right)\right)$; they are then passed through the source-domain generator $G_{X}$ to produce reconstructed sample $\hat{X}=\left(G_{X}\left(\hat{Y}\right)\right)$. Similarly, the target-domain data *Y* are translated into $\hat{X}=\left(G_{X}\left(Y\right)\right)$ and then mapped back to the target domain as $\hat{Y}=\left(G_{Y}\left(\hat{X}\right)\right)$. The loss is computed by measuring the $l_{1}$ norm between the original input and the reconstructed sample:

$$L_{cycle}\left(G_{X},G_{Y}\right)=E_{X∼P(X)}\left[{∥G_{X}\left(G_{Y}\left(X\right)\right)-X∥}_{1}\right]+E_{Y∼P(Y)}\left[{∥G_{Y}\left(G_{X}\left(Y\right)\right)-Y∥}_{1}\right]$$

This formulation penalizes discrepancies between the input and its reconstructed version, even in the absence of paired training data. By enforcing this cyclic constraint, the model preserves gesture-related features across domain transformations, reducing the dependence on labeled target samples and enhancing cross-subject generalization.

Conditional Vector Loss. To overcome the spatial resolution limitations of WiFi signals and enhance the structural integrity of the generated data, this framework introduces a conditional vector loss guided by vision-based skeletal features. Specifically, 3D skeletal joint positions extracted from vision data are used to construct spatial matrices that capture user-specific biomechanical information. Let the coordinates of the *i*th joint be $\left(x_{i},y_{i},z_{i}\right)$, where $i\in \left\{1,2,\ldots ,17\right\}$. The spatial relationship matrices along the *x*, *y*, and *z* axes are constructed as

$$M_{k}\left(i,j\right)=\left\{\begin{array}{c} {\left(k_{i}-k_{j}\right)}^{2},i\neq j\\ k_{i}^{2},i=j \end{array}\right.,k\in \left\{x,y,z\right\}$$

where $M_{k}$ represents the 17×17 matrix for dimension *k*, and each element quantifies the squared Euclidean distance between joint *i* and joint *j* along this axis. These matrices encode the spatial configuration of the human body during gesture execution, providing high-resolution semantic priors for cross-modal synthesis.

To align the structural features of the generated WiFi data with the target user’s biomechanics, the conditional vector loss computes the mean squared error (MSE) between the corresponding spatial matrices of the source and target users:

$$L_{cond}=\frac{1}{3}\sum_{k\in \left\{x,y,z\right\}}{∥M_{k}^{s}-M_{k}^{t}∥}_{2}^{2}$$

where $M_{k}^{s}$ and $M_{k}^{t}$ denote the source- and target-domain matrices along dimension *k*. The loss functions as a cross-modal supervisory signal, driving the generator to produce user-specific WiFi data consistent with the spatial dynamics in the vision input. This enables zero-shot generalization while eliminating the need for additional WiFi data from target users.

The overall loss function of the proposed CM-GR framework integrates adversarial learning, cycle consistency, and cross-modal conditioning:

$$\begin{array}{cc} L_{total}\left(G_{X},G_{Y},D_{X},D_{Y}\right) & =L_{GAN}\left(G_{Y},D_{Y},X,Y\right)+L_{GAN}\left(G_{X},D_{X},Y,X\right)\\ \; & +\;\lambda_{1}L_{cycle}\left(G_{X},G_{Y}\right)+\lambda_{2}L_{cond}\left(X,Y\right) \end{array}$$

In this formulation, adversarial components promote domain-specific generation by training discriminators to distinguish real from synthesized data, while generators learn to fool them. The cycle consistency loss encourages structural preservation by requiring that data mapped to the opposite domain and back retain their original semantic content. The conditional vector loss, derived from vision-based skeletal differences, guides the synthesis toward user-specific characteristics by embedding cross-modal priors into the generation process. Hyperparameters $\lambda_{1}$ and $\lambda_{2}$ balance the contributions of the loss terms, where $\lambda_{1}$ emphasizes semantic alignment and $\lambda_{2}$ enhances cross-user generalizability. Their values are determined empirically through grid search on the validation set, and we found that the chosen settings yield stable performance while avoiding overemphasis on either objective.

### 4.4. Gesture Recognition Module

To enhance generalization in cross-subject gesture recognition, the proposed CM-GR framework first synthesizes WiFi data that capture the gesture-specific dynamics of new target users. These synthesized data serve as input to the gesture recognition module, which performs hierarchical feature extraction and classification. As detailed in Table 3, the feature extractor consists of convolutional layers (Conv2D), pooling operations (MaxPooling2D), and dropout regularization to capture robust spatial–temporal patterns. The classifier is composed of three fully connected layers (Dense) and a final Softmax activation for gesture classification. With domain-adaptive synthetic data, the model avoids costly user-specific data collection while achieving the reliable recognition of gestures from unseen subjects.

## 5. Evaluation

### 5.1. Experimental Setup and Parameter Settings

Given the limited availability of public datasets offering synchronized WiFi and video data, this study uses both the publicly available MM-Fi [44] dataset and a newly collected multimodal dataset named SelfSet, as summarized in Table 4. The MM-Fi dataset comprises five sensing modalities, namely RGB video, depth video, WiFi CSI, LiDAR point clouds, and millimeter-wave radar. It records 27 activities performed by 10 volunteers across four distinct environments (E1–E4). For this work, only the RGB video and WiFi CSI modalities are utilized. Among the 27 activities, nine purely gesture-based actions are selected, namely “horizontal arm stretch”, “vertical arm stretch”, “left arm extension”, “right arm extension”, “simultaneous arm extension”, “left arm raise”, “right arm raise”, “left hand wave”, and “right hand wave”.

**Figure 6:**
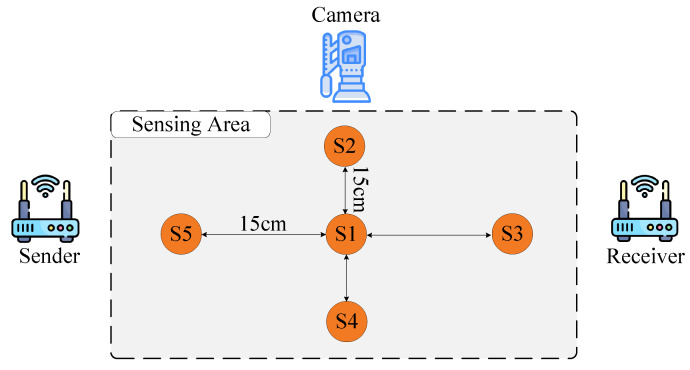
Experimental setup of the SelfSet dataset collection, illustrating the five spatial locations (S1–S5) considered during gesture recording.

To address the limitations of existing datasets in supporting personalized gesture generation, we constructed SelfSet, a multimodal dataset comprising synchronized WiFi CSI and RGB video data. Ten participants performed six distinct gestures, including “push”, “pull”, “slide”, “sweep”, “throw”, “draw a circle”, and “draw zigzag”. As shown in Figure 6, each gesture was performed from five predefined spatial locations (S1–S5) to capture gesture variability across different spatial contexts. Each gesture was repeated 30 times. RGB videos were captured using a Sony FDR-AX60 camera (Sony Corporation, Tokyo, Japan), while WiFi CSI data were collected using two computers equipped with Intel 5300 NICs (Intel Corporation, Santa Clara, CA, USA) functioning as the transmitter and receiver, respectively. Both devices utilized three antennas, resulting in 3 × 3 × 30 = 270 subcarriers for each WiFi sample. An overview of the participants and their characteristics is provided in Table 5.

To evaluate the effectiveness of the proposed CM-GR framework, experiments are conducted under several settings. First, we compare the performance with and without the data generation module to assess its contribution to cross-subject gesture recognition. Second, we evaluate the quality of the generated data. Third, CM-GR is compared with three representative generative models, namely DCGAN [45], CsiGAN [12], and RF-Diffusion [13], which are selected based on their relevance and performance in RF data synthesis. In addition, we further compare CM-GR with WiGr [46], a non-generative cross-domain WiFi gesture recognition system that constructs a domain-transferable embedding space using a dual-path prototypical network. This baseline is included to provide a stronger comparison with state-of-the-art recognition methods that rely on domain-invariant feature learning rather than data generation. Finally, an ablation study is conducted to evaluate the contribution of the cross-modal conditional vector to the overall performance of the CM-GR framework. It is worth noting that cross-modal learning in this work is conducted within individual datasets, and separate models are trained on MM-Fi and SelfSet to ensure a fair and independent evaluation.

All experiments were conducted on a server running Ubuntu 20.04 with Python 3.8.19, PyTorch 2.0.1, and CUDA 11.8. The hardware configuration consisted of an Intel Xeon Gold 6226 CPU (Intel Corporation, Santa Clara, CA, USA), 256 GB RAM (Kingston Technology Corporation, Fountain Valley, CA, USA), and two NVIDIA RTX 4090 GPUs (NVIDIA Corporation, Santa Clara, CA, USA), although only one GPU was utilized for both training and testing. Under this setup, model training typically required approximately five minutes, while inference across the test set was completed in less than one minute.

### 5.2. Overall Performance

**Figure 7:**
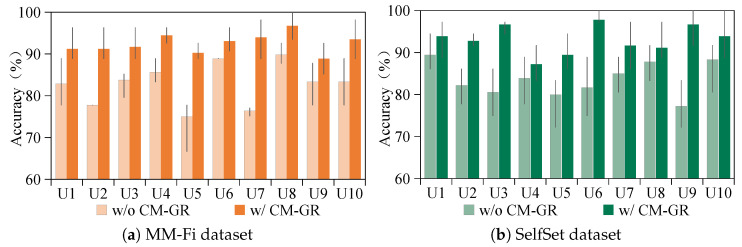
Cross-subject gesture recognition performance of CM-GR on MM-Fi and SelfSet datasets.

To evaluate the impact of the proposed CM-GR on cross-subject gesture recognition using WiFi sensing, experiments were conducted by treating each volunteer as an unseen target user. As illustrated in Figure 7, yellow bars denote results on the MM-Fi dataset and green bars on the SelfSet dataset, with min–max lines showing the accuracy range across ten repeated runs. U1–U10 correspond to the ten participants. The results demonstrate that CM-GR significantly enhances the cross-subject performance on both datasets. In particular, the maximum accuracy improvement reaches 10.26% on MM-Fi and 9.5% on SelfSet, highlighting its effectiveness in generating user-specific WiFi gesture data for robust cross-subject generalization.

**Figure 8:**
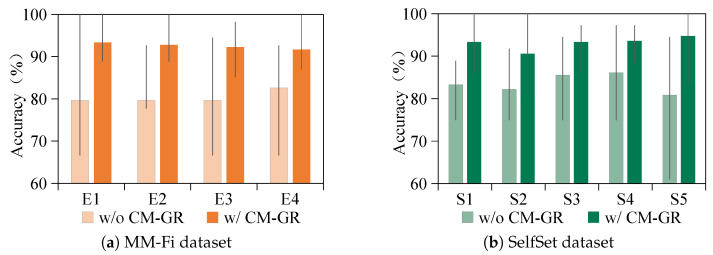
Performance of cross-subject gesture recognition across different environments or locations.

Additionally, the recognition performance was assessed across varying environments and spatial locations. WiFi-based sensing captures signal variations caused by multipath propagation effects such as reflection and diffraction, particularly from human movement. However, the environmental conditions and user location can significantly affect signal behavior. For example, reflections from static objects such as furniture, walls, and ceilings introduce variability into the received signals. To evaluate the generalization performance of the model under these variations, Figure 8 presents the recognition accuracy across different environments and locations in the MM-Fi and SelfSet datasets. In this figure, yellow and green bars correspond to MM-Fi and SelfSet results, respectively. The min–max line represents the accuracy range observed when different users are treated as unseen subjects under the same environmental or spatial setting. MM-Fi environments are labeled E1–E4, while SelfSet locations are denoted as S1–S5. The results show that CM-GR consistently outperforms the baseline configuration without data generation, with an average accuracy gain of 9.29%. An exception is observed in environment E1, where the baseline model already achieves 100% accuracy. This could be due to E1 being a relatively open space with minimal static interference and high user similarity in body size and shape, combined with the fact that MM-Fi gestures involve large-scale arm movements. Despite this outlier, CM-GR achieves higher accuracy in all other environments and locations, with the maximum improvement reaching 13.89%. These findings further validate CM-GR’s capability to enhance WiFi-based cross-subject gesture recognition under diverse real-world conditions.

### 5.3. Performance of Data Generation

**Figure 9:**
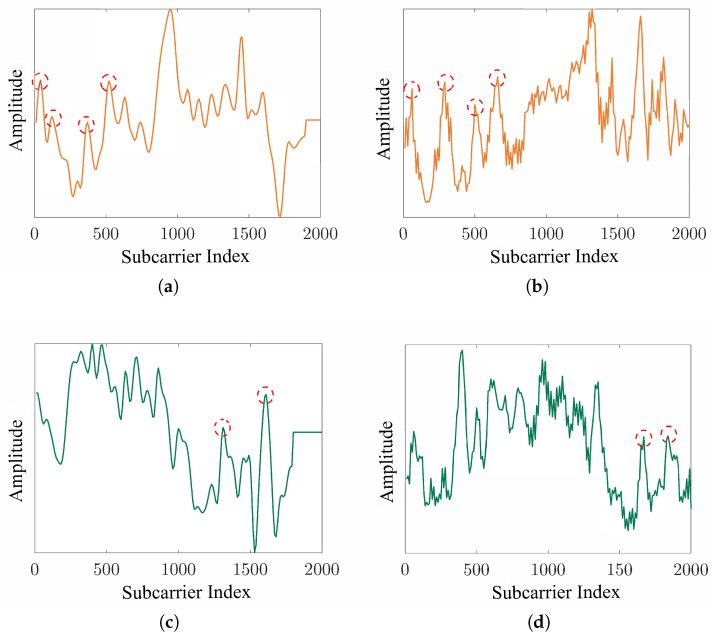
Amplitude variations of real and generated data. (**a**) Real WiFi data of user U1. (**b**) Generated WiFi data for user U1. (**c**) Real WiFi data of user U2. (**d**) Generated WiFi data for user U2.

**Figure 10:**
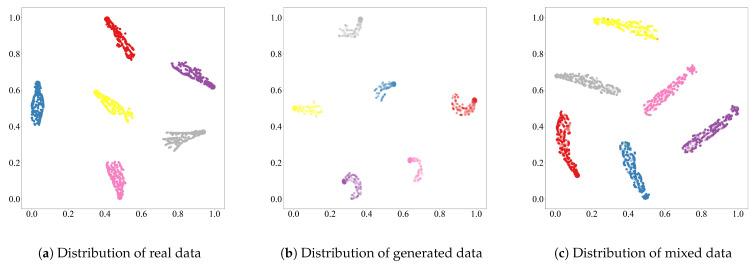
Comparison of data distributions for real data, generated data, and mixed data. Different colors indicate different gesture categories.

**Figure 11:**
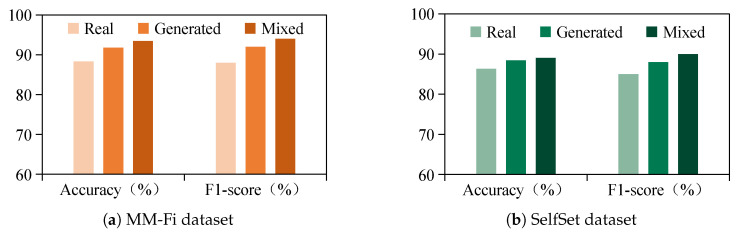
Comparison of real, generated, and mixed data on MM-Fi and SelfSet with accuracy and F1-score.

To assess the performance of the proposed data generation module, we conduct a series of experiments involving visual, distributional, and classification-based evaluations. First, we compare the amplitude variations of real and generated WiFi signals to evaluate their similarity, as shown in Figure 9. Second, we apply t-SNE dimensionality reduction to visualize the distributions of real, generated, and mixed data, thereby evaluating the alignment between generated samples and real data (Figure 10). Finally, we evaluate the gesture recognition performance using five-fold cross-validation for each data type, with both the accuracy and F1-score reported to provide a more comprehensive assessment, as illustrated in Figure 11.

Figure 9 presents samples from the SelfSet dataset for users U1 and U2, including both their real WiFi data and the cross-subject generated versions. The generated signals exhibit strong similarity to the temporal patterns of real signals. As shown in Figure 9a,b, U1’s data display a higher amplitude in the latter half and four distinct peaks in the earlier segment, with the generated signals closely matching the real ones. Similarly, Figure 9c,d demonstrate that U2’s signals show a higher amplitude in the first half and two clear peaks in the latter segment for both the real and generated data.

To further analyze the quality of the generated samples, we project the real, generated, and mixed datasets into a two-dimensional space using t-SNE. As shown in Figure 10, each color represents a different gesture class in the SelfSet dataset. Figure 10a,b illustrate that generated data form clear, gesture-specific clusters consistent with real data distributions. When combined with real samples (Figure 10c), these clusters remain compact, demonstrating that the generated data are both distributionally aligned and semantically discriminative.

Figure 11 compares the gesture recognition performance across the MM-Fi and SelfSet datasets using real, generated, and mixed data, evaluated by both the accuracy and F1-score. The results demonstrate that the mixed dataset consistently achieves the highest performance on both metrics, followed by the generated data and then the real data alone. This consistent trend across the accuracy and F1-score confirms that the proposed module generates high-quality synthetic samples and enhances the training diversity when combined with real data. It thereby reduces the data collection effort and improves the robustness in cross-subject recognition.

### 5.4. Comparisons with Existing Methods

To evaluate the performance of the proposed CM-GR framework, we compare it against three representative data generation methods—DCGAN [45], CsiGAN [12], and RF-Diffusion [13]—as well as WiGr [46], a state-of-the-art non-generative cross-domain WiFi gesture recognition system. DCGAN is chosen for its compatibility with the convolution-based architectures commonly used in WiFi gesture recognition. CsiGAN is the first approach to apply GANs for WiFi signal generation, while RF-Diffusion represents a recent advancement that introduces diffusion models into RF signal synthesis. WiGr constructs a domain-transferable embedding space via a dual-path prototypical network, providing a strong non-generative baseline for cross-domain recognition.

**Figure 12:**
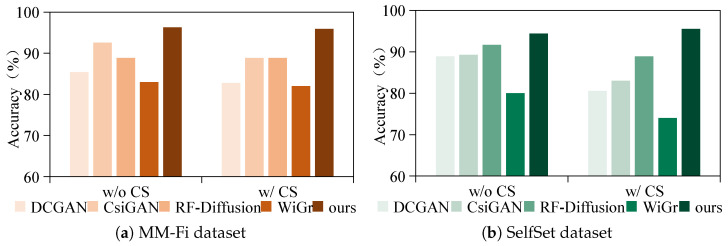
Comparison of CM-GR with generative baselines (DCGAN, CsiGAN, RF-Diffusion) and a non-generative baseline (WiGr) on the MM-Fi and SelfSet datasets.

Figure 12 illustrates the recognition accuracy of the five methods on both the MM-Fi and SelfSet datasets under two settings, namely without cross-subject (w/o CS) and with cross-subject (w/ CS) recognition. In the w/o CS setting, the accuracy is computed using five-fold cross-validation. In the w/ CS setting, each participant is treated as an unseen target user, and the final result is the average accuracy across all users. Yellow and green bars indicate results for the MM-Fi and SelfSet datasets, respectively. The results show that CM-GR consistently outperforms all baseline methods in both settings and across both datasets. On the MM-Fi dataset, CM-GR achieves up to 13.13%, 7.04%, and 7.04% higher cross-subject recognition accuracy compared to DCGAN, CsiGAN, and RF-Diffusion, respectively, and outperforms WiGr by 13.93%. Similarly, on the SelfSet dataset, CM-GR surpasses the same generative baselines by 14.99%, 12.52%, and 6.66%, and achieves an improvement of 21.55% over WiGr. These findings confirm that CM-GR achieves enhanced generalization and synthesis quality, enabling robust cross-subject gesture recognition in WiFi sensing.

**Figure 13:**
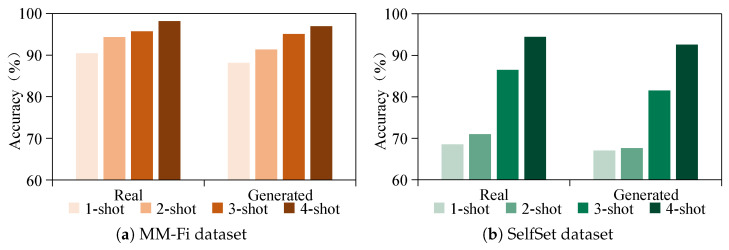
Comparison of few-shot (1–4 shots) gesture recognition on MM-Fi and SelfSet datasets using real and generated data.

To further evaluate the effectiveness of the proposed CM-GR framework, we compare it with existing few-shot learning settings on both the MM-Fi and SelfSet datasets. As shown in Figure 13, experiments are conducted under one-shot, two-shot, three-shot, and four-shot configurations using both real and generated data. The results demonstrate that generated data achieve recognition performance comparable to that of real data across all settings. This finding validates the effectiveness of the proposed generation strategy in producing high-quality synthetic samples and supporting few-shot learning. More importantly, the comparable performance between real and generated samples suggests that our approach is capable of approximating zero-shot learning, thereby reducing the need for costly data collection in practical deployments.

### 5.5. Performance of Cross-Modal Semantic Learning

**Figure 14:**
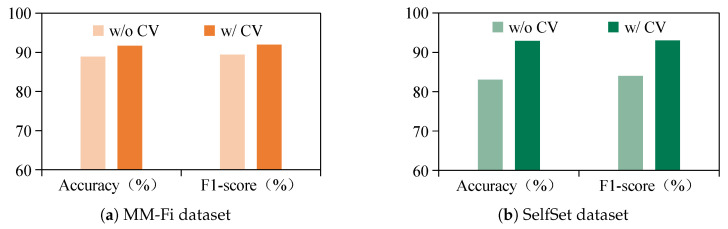
Ablation study results when comparing models with and without conditional vectors, evaluated by accuracy and F1-score on MM-Fi and SelfSet datasets.

Beyond employing generative adversarial networks, the proposed CM-GR framework integrates cross-modal semantic learning to construct conditional vectors, enhancing the fidelity and relevance of the generated WiFi data. To assess the contribution of conditional vectors, an ablation study is conducted under cross-subject settings. The model is evaluated in two configurations: without conditional vectors (w/o CV) and with conditional vectors (w/ CV). As illustrated in Figure 14, the w/ CV setting demonstrates superior recognition performance on both datasets, evaluated in terms of the accuracy and F1-score. Specifically, the accuracy improves by 2.78% on the MM-Fi dataset and 9.86% on the SelfSet dataset, with the F1-scores improving by 2.6% and 9%, respectively. These results confirm the effectiveness of introducing target user characteristics through cross-modal conditional vectors, which guide the generator in synthesizing user-specific WiFi data and enhance generalization to unseen subjects.

## 6. Conclusions

This paper proposes CM-GR, a cross-modal gesture recognition framework that synthesizes user-specific WiFi data guided by visual priors. By embedding 3D skeletal joint information into spatial matrices and constructing dynamic conditional vectors, CM-GR incorporates biomechanical constraints that tailor signal generation to individual users. A dual-stream adversarial architecture further enables bidirectional domain mapping between the source and target domains, eliminating the need for costly per-user WiFi data collection. Extensive evaluations on the MM-Fi and SelfSet datasets demonstrate the effectiveness of CM-GR. The framework achieves up to 15% higher cross-subject recognition accuracy compared with state-of-the-art generative baselines, and it further outperforms non-generative systems and few-shot learning approaches. These results validate the advantage of leveraging cross-modal semantic learning to bridge the gap between visual kinematics and RF signal semantics, yielding robust and user-adaptive gesture recognition. Although CM-GR introduces moderate computational overhead, the framework remains efficient in practice, with training requiring only a few minutes and inference completed in less than 1 min on modern GPU systems. Moreover, it is amenable to optimization through pruning, quantization, and hardware acceleration, which will be the focus of future work to support scalable real-time deployment in ubiquitous computing environments.

## Figures and Tables

**Table 1:** Network architecture of the generator, including the downsampling, residual, and upsampling modules.

Layer Index	Operation Type	Configuration
1	Conv2D	Kernel = 7 × 7, FM = 32, Stride = 1, BN, ReLU
2	Conv2D	Kernel = 3 × 3, FM = 64, Stride = 2, BN, ReLU
3	Conv2D	Kernel = 3 × 3, FM = 128, Stride = 2, BN, ReLU
4	Residual Block × 9	Kernel = 3 × 3, FM = 128, Stride = 1, BN, ReLU
5	Deconv2D	Kernel = 3 × 3, FM = 64, Stride = 2, BN, ReLU
6	Deconv2D	Kernel = 3 × 3, FM = 32, Stride = 2, BN, ReLU

**Table 2:** Network architecture of the discriminator.

Layer Index	Operation Type	Configuration
1	Conv2D	Kernel = 4 × 4, FM = 64, Stride = 2, LeakyReLU
2	Conv2D	Kernel = 4 × 4, FM = 128, Stride = 2, BN, LeakyReLU
3	Conv2D	Kernel = 4 × 4, FM = 256, Stride = 2, BN, LeakyReLU
4	Conv2D	Kernel = 4 × 4, FM = 512, Stride = 1, BN, LeakyReLU
5	Conv2D	Kernel = 4 × 4, FM = 1, Stride = 1

**Table 3:** Network architecture of the gesture recognition module.

Layer Index	Operation Type	Configuration
1	Conv2D	Kernel = 2 × 2, FM = 64, Stride = 1, ReLU
2	MaxPooling2D	PoolSize = 3 × 3, BN
3	Dropout	*p* = 0.5
4	Conv2D	Kernel = 2 × 2, FM = 64, Stride = 1, ReLU
5	MaxPooling2D	PoolSize = 3 × 3, BN1
6	Dropout	*p* = 0.5
7	Conv2D	Kernel = 1 × 1, FM = 32, Stride = 1, BN, ReLU
8	Dense	FM = 1280, BN, ReLU
9	Dense	FM = 128, BN, ReLU
10	Dense	FM = 6, Softmax

**Table 4:** Summary of the two datasets used for evaluation (# indicates the number of items).

Dataset Name	# Gestures	# Volunteers	# Envs	Env IDs
MM-Fi [44]	9	10	4	E1, E2, E3, E4
SelfSet	6	10	5	S1, S2, S3, S4, S5

**Table 5:** Overview of participants and their characteristics in the SelfSet dataset.

Participant ID	Gender	Age	Height (m)	Weight (kg)
U1	Male	24	1.69	66
U2	Female	29	1.63	48
U3	Female	25	1.61	51
U4	Male	26	1.77	71
U5	Male	23	1.70	62
U6	Male	24	1.76	69
U7	Male	25	1.80	73
U8	Female	24	1.65	46
U9	Male	22	1.68	59
U10	Female	23	1.73	61

## Data Availability

The data presented in this study are available upon request from the corresponding author.
